# Supplementary material for: Selection for immune evasion in SARS-CoV-2 revealed by high-resolution epitope mapping and sequence analysis
Source: iScience. 2023 Jul 13;26(8):107394. doi: 10.1016/j.isci.2023.107394 (PMC10433132; doi:10.1016/j.isci.2023.107394)
Supplement: Document S1. Figures S1–S8 and Tables S1 and S12 [file mmc1.pdf]

iScience, Volume *26*

Supplemental information

Selection for immune evasion in SARS-CoV-2 revealed by high-resolution epitope mapping and sequence analysis

Arnaud N'Guessan, Senthilkumar Kailasam, Fatima Mostefai, Raphaël Poujol, Jean-Christophe Grenier, Nailya Ismailova, Paola Contini, Raffaele De Palma, Carsten Haber, Volker Stadler, Guillaume Bourque, Julie G. Hussin, B. Jesse Shapiro, Jörg H. Fritz, and Ciriaco A. Piccirillo

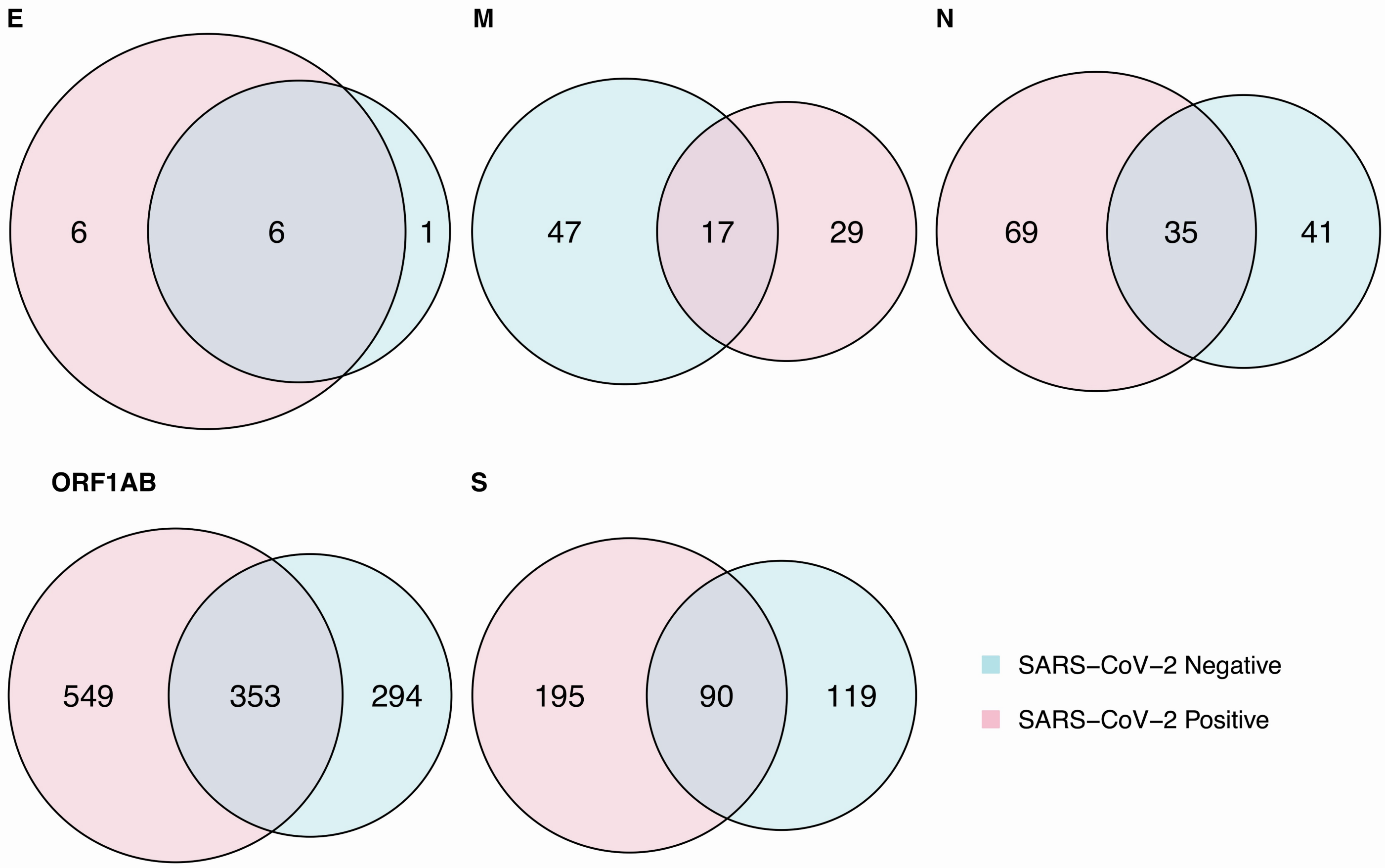
E
6
6
1
M
47
17
29
N
69
35
41
ORF1AB
549
353
294
S
195
90
119
SARS−CoV−2 Negative
SARS−CoV−2 Positive

Figure S1

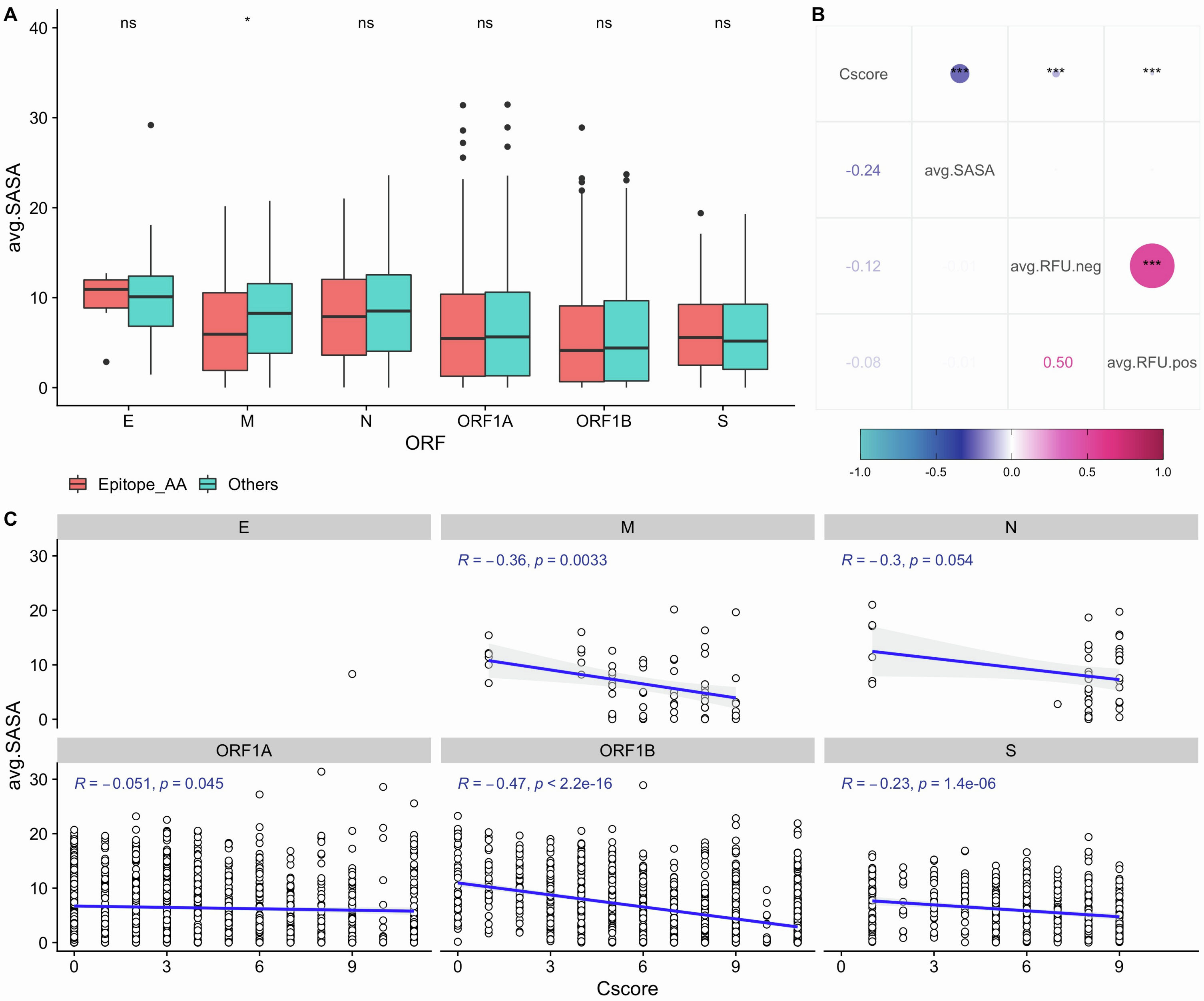
A
B
C
ns
*
avg.SASA
ORF
E
M
N
ORF1A
ORF1B
S
Epitope_AA
Others
Cscore
avg.SASA
avg.RFU.neg
avg.RFU.pos
-0.24
-0.12
-0.08
0.50
***
-1.0
-0.5
0.0
0.5
1.0
R = −0.36, p = 0.0033
R = −0.3, p = 0.054
R = −0.051, p = 0.045
R = −0.47, p < 2.2e-16
R = −0.23, p = 1.4e-06

Figure S2

ORF1A

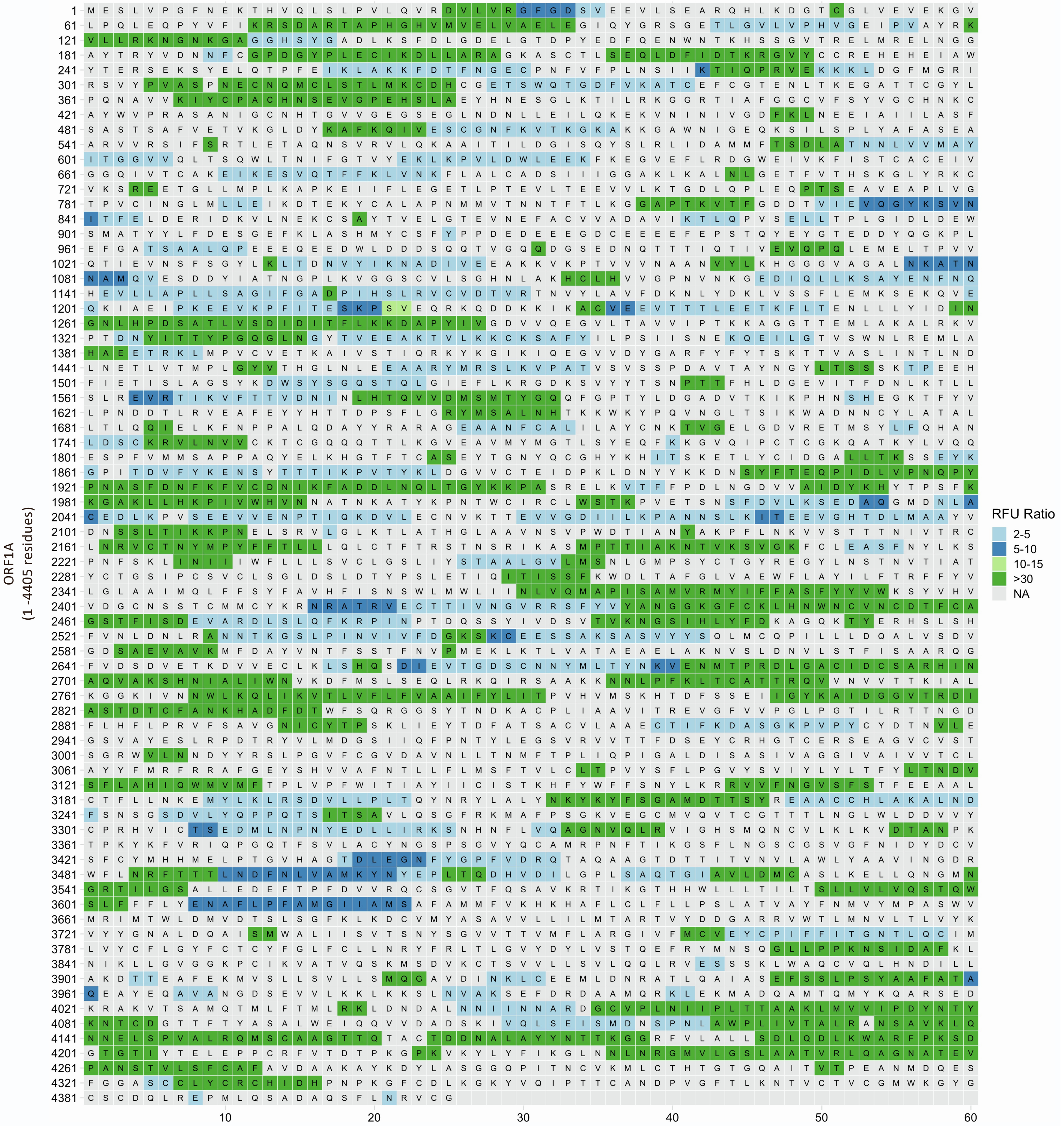

Figure S3

ORF1B

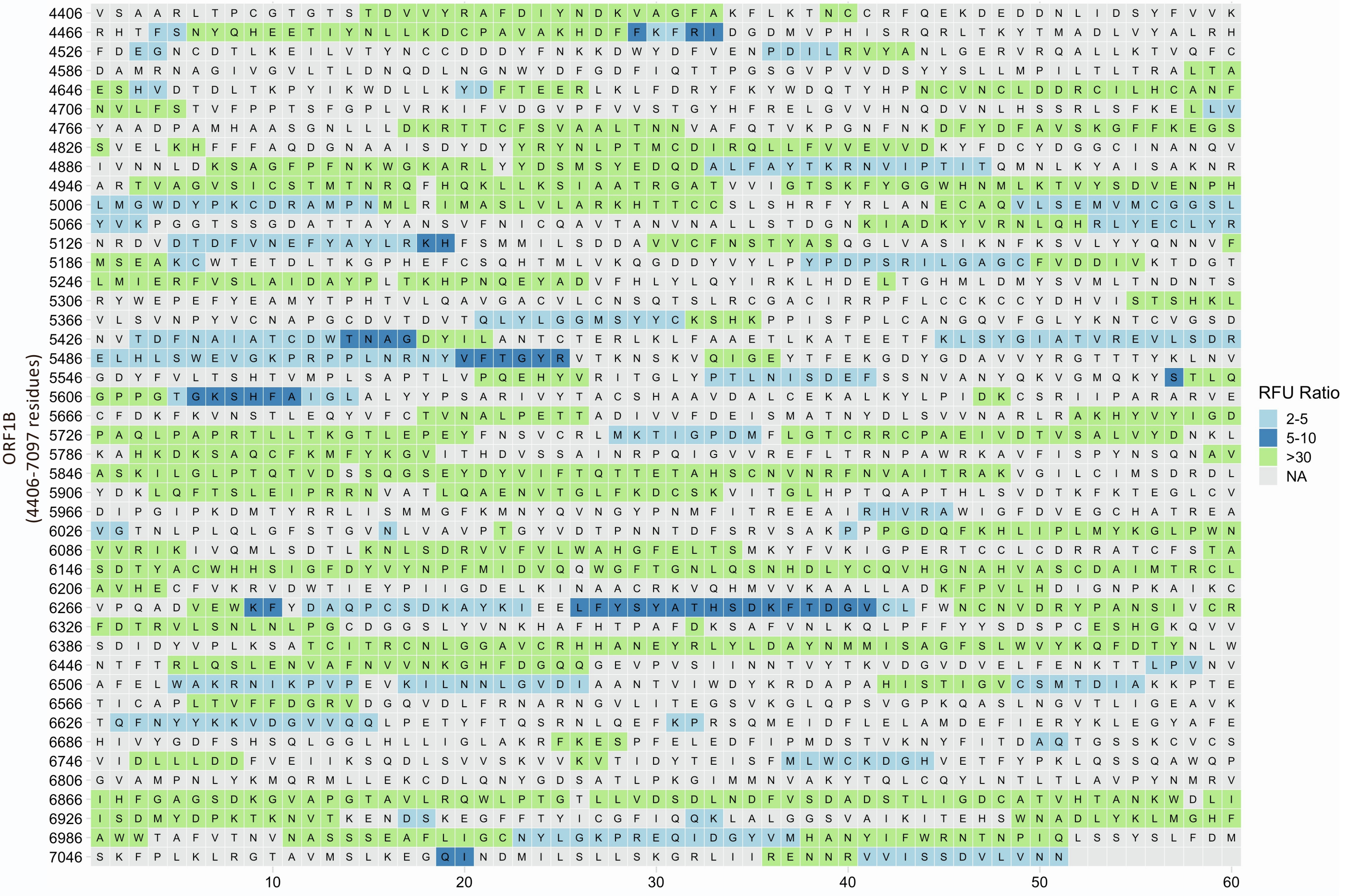

Figure S4

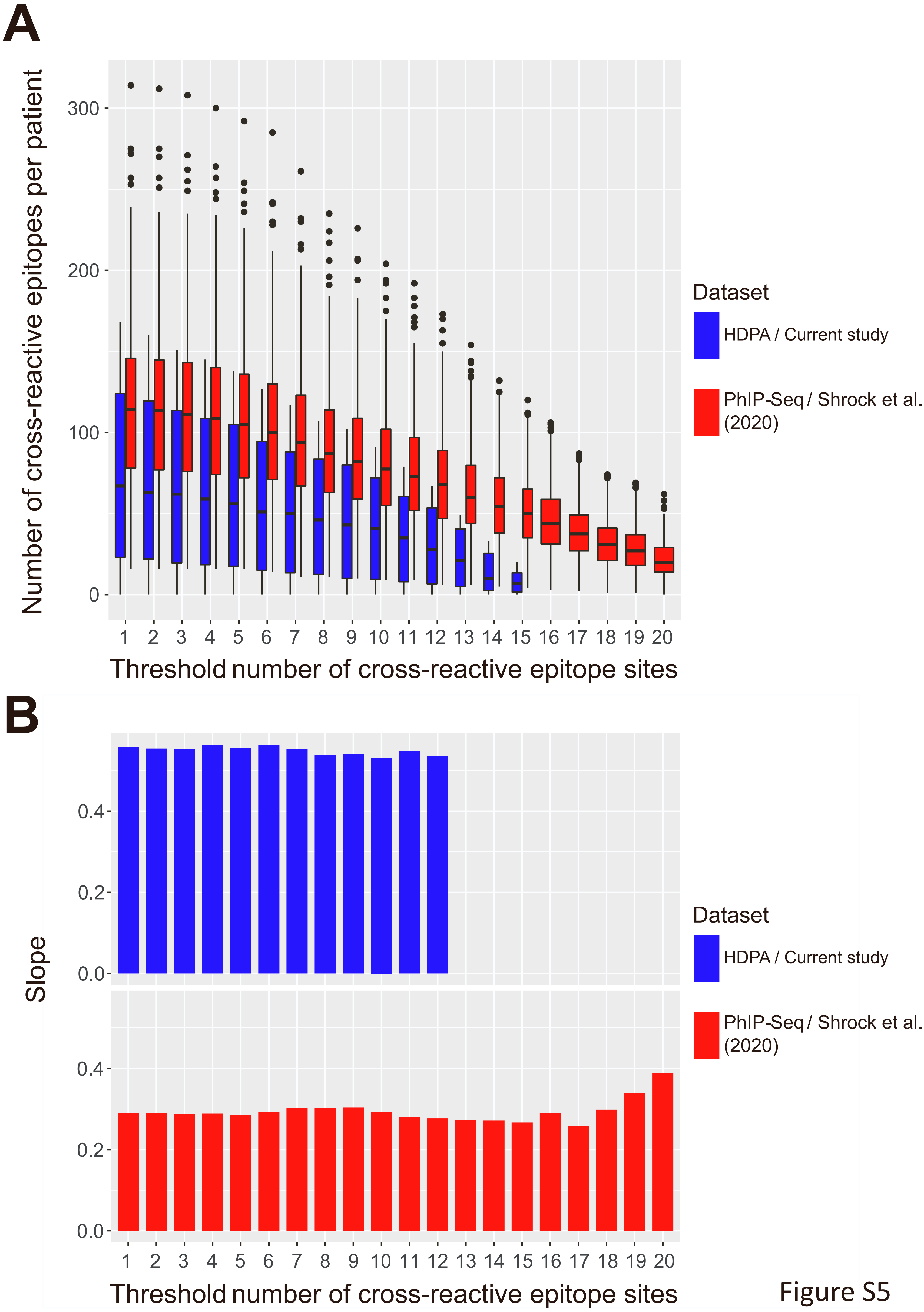

A
Number of cross-reactive epitopes per patient
300
200
100
0
1
2
3
4
5
6
7
8
9
10
11
12
13
14
15
16
17
18
19
20
Threshold number of cross-reactive epitope sites
Dataset
HDPA / Current study
PhIP-Seq / Shrock et al. (2020)
B
Slope
0.4
0.2
0.0
0.4
0.2
0.0
1
2
3
4
5
6
7
8
9
10
11
12
13
14
15
16
17
18
19
20
Threshold number of cross-reactive epitope sites
Dataset
HDPA / Current study
PhIP-Seq / Shrock et al. (2020)

Figure S5

Figure S6

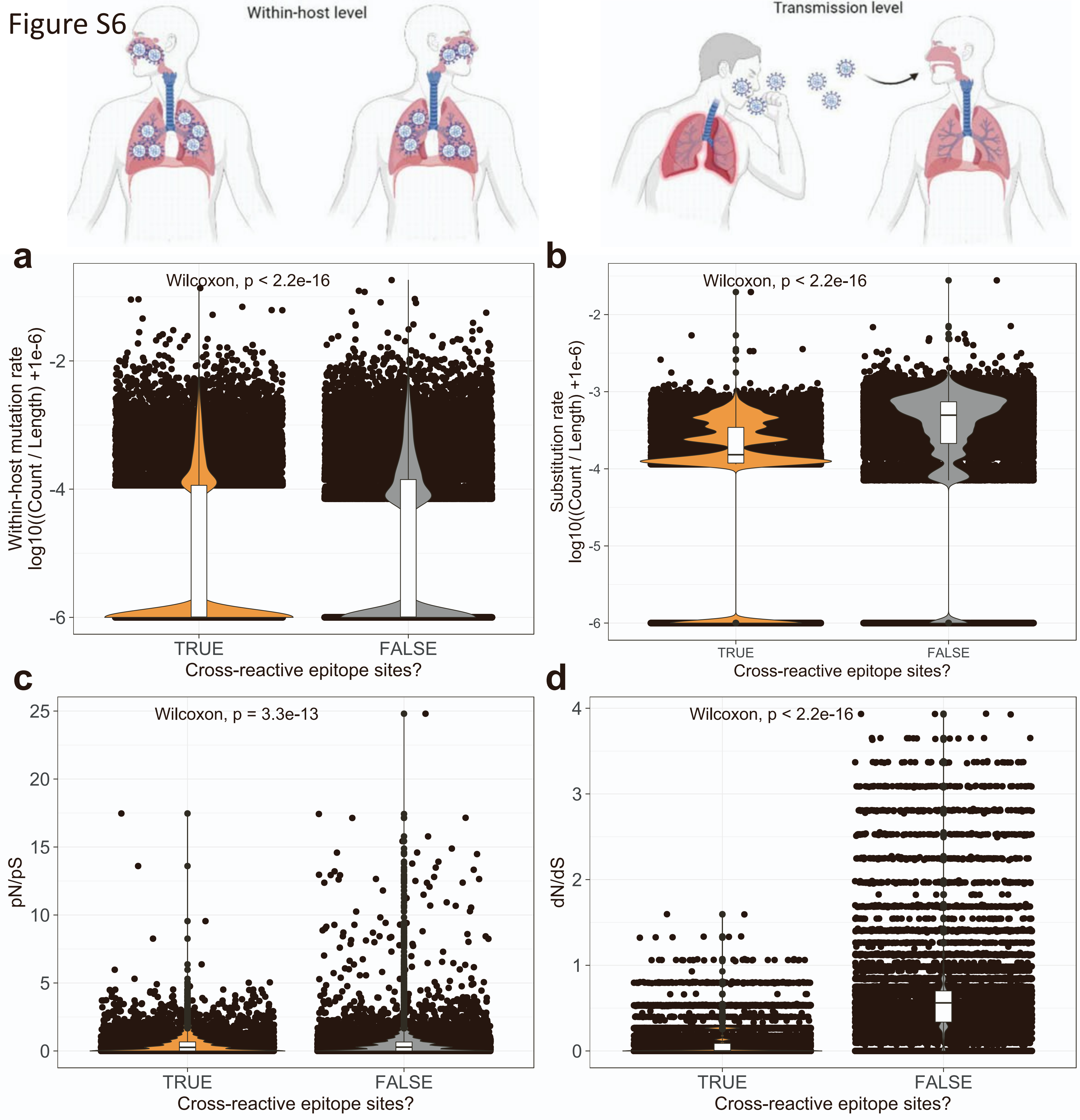
Within-host level
Transmission level
a
Wilcoxon, p < 2.2e-16
Within-host mutation rate
log10((Count / Length) +1e-6)
-2
-4
-6
TRUE
FALSE
Cross-reactive epitope sites?
b
Wilcoxon, p < 2.2e-16
Substitution rate
log10((Count / Length) +1e-6)
-2
-3
-4
-5
-6
TRUE
FALSE
Cross-reactive epitope sites?
c
Wilcoxon, p = 3.3e-13
pN/pS
25
20
15
10
5
0
TRUE
FALSE
Cross-reactive epitope sites?
d
Wilcoxon, p < 2.2e-16
dN/dS
4
3
2
1
0
TRUE
FALSE
Cross-reactive epitope sites?

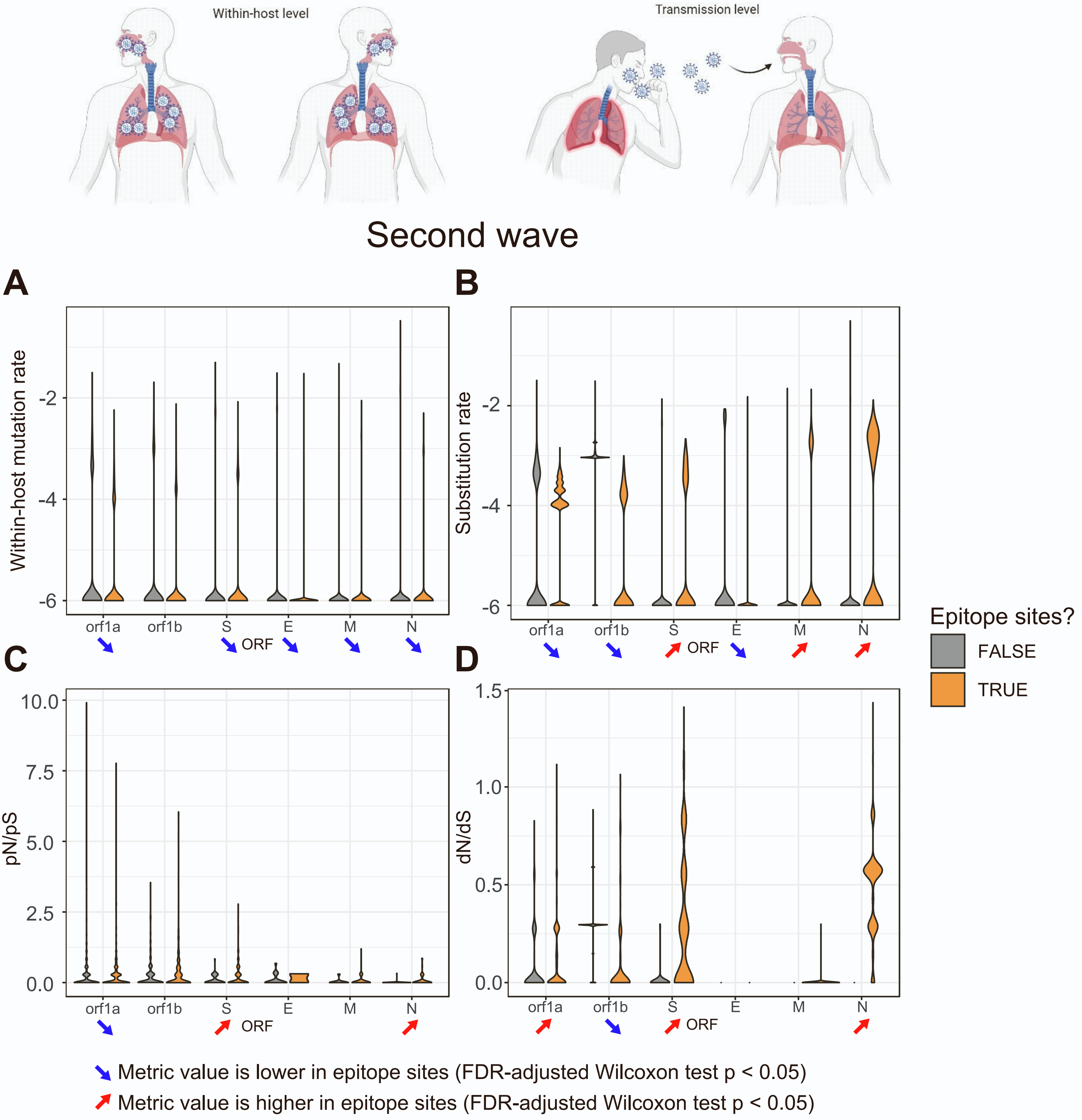

Within-host level
Transmission level
Second wave
A
B
C
D
Within-host mutation rate
Substitution rate
pN/pS
dN/dS
orf1a
orf1b
S
E
M
N
ORF
Epitope sites?
FALSE
TRUE
Metric value is lower in epitope sites (FDR-adjusted Wilcoxon test p < 0.05)
Metric value is higher in epitope sites (FDR-adjusted Wilcoxon test p < 0.05)

Figure S7

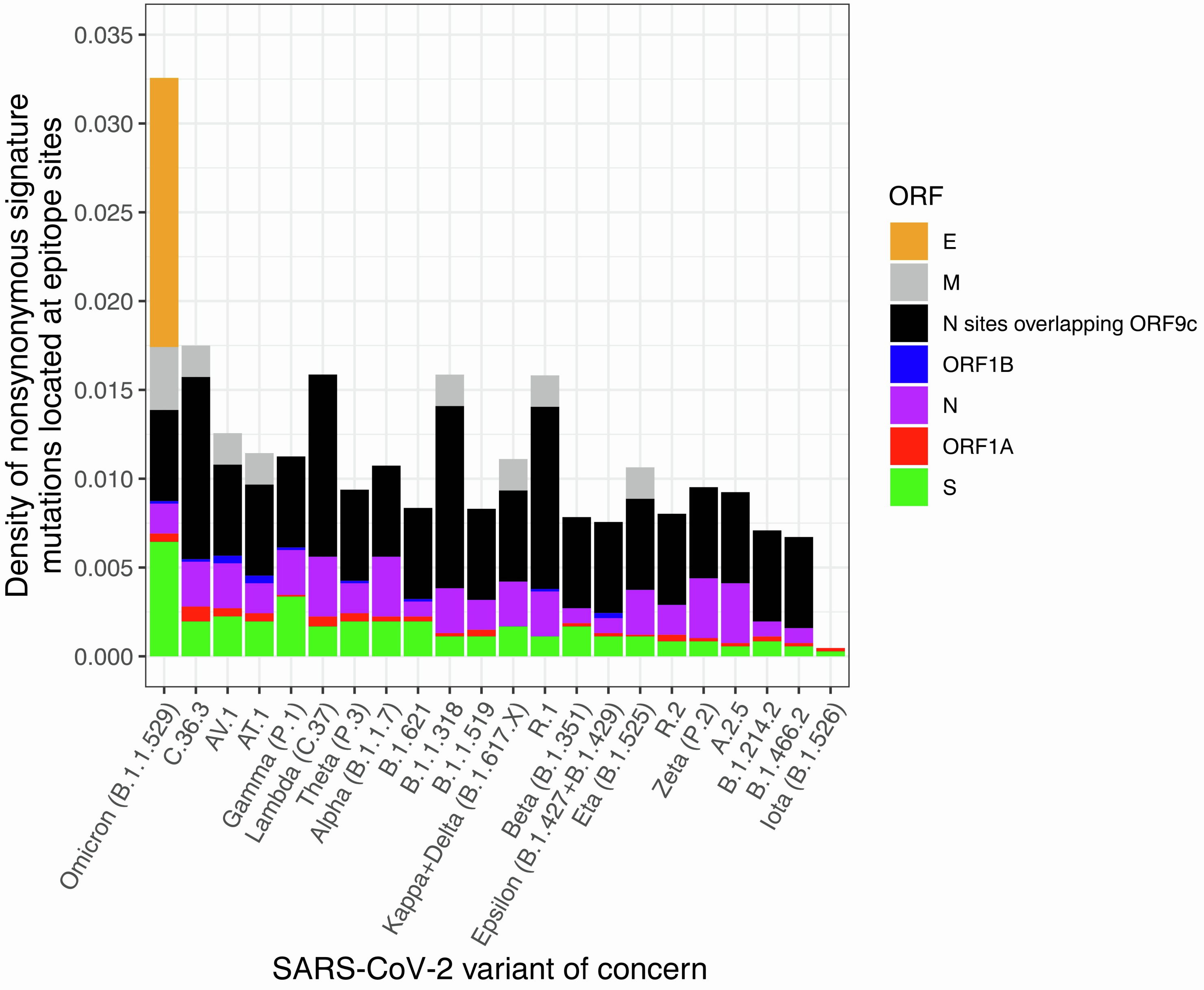

0.035
0.030
0.025
0.020
0.015
0.010
0.005
0.000
Density of nonsynonymous signature mutations located at epitope sites
Omicron (B.1.1.529)
C.36.3
AV.1
AT.1
Gamma (P.1)
Lambda (C.37)
Theta (P.3)
Alpha (B.1.1.7)
B.1.621
B.1.1.318
B.1.1.519
Kappa+Delta (B.1.617.X)
R.1
Beta (B.1.351)
Epsilon (B.1.427+B.1.429)
Eta (B.1.525)
R.2
Zeta (P.2)
A.2.5
B.1.214.2
B.1.466.2
Iota (B.1.526)
SARS-CoV-2 variant of concern
ORF
E
M
N sites overlapping ORF9c
ORF1B
N
ORF1A
S

Figure S8

SARS-CoV2-specific Peptides

	Spike	Nucleocapsid	Envelope	Membrane	ORF1ab	TOTAL
**SARS-CoV-2 negative**	119	41	1	47	294	502
**SARS-CoV-2 positive**	195	69	6	29	549	848
**Overlap**	90	35	6	17	353	501
**Total**	404	145	13	93	1196	1851

Table 1

Name	Genus	Subgenus	Taxonomy ID	ORF1AB	Spike	Envelope	Membrane	Nucleocapsid
SARS-CoV-2	Betacoronavirus	Sarbecovirus	NCBI:txid2697049	YP_009724389.1	YP_009724390.1	YP_009724392.1	YP_009724393.1	YP_009724397.2
229E	Alphacoronavirus	Duvinacovirus	NCBI:txid11137	ARU07599.1	ARU07601.1	ARU07603.1	ARU07604.1	ARU07605.1
NL63	Alphacoronavirus	Setracovirus	NCBI:txid277944	YP_003766.2	YP_003767.1	YP_003769.1	YP_003770.1	YP_003771.1
OC43	Betacoronavirus	Embecovirus	NCBI:txid31631	YP_009555238.1	YP_009555241.1	YP_009555243.1	YP_009555244.1	YP_009555245.1
HKU1	Betacoronavirus	Embecovirus	NCBI:txid290028	YP_173236.1	YP_173238.1	YP_173240.1	YP_173241.1	YP_173242.1

Table S12

SUPPLEMENTARY INFORMATION

Figure S1. Numbers of SARS-CoV-2-specific epitope-defining peptides identified by HDPA, related to Figure 1. Number of SARS-CoV-2-specific epitopes identified with high density peptide arrays (HDPA) in spike (S) protein, envelope (E) protein, membrane (M) glycoprotein, nucleocapsid (N) phosphoprotein, and ORF1AB. Number of unique peptides that showed a significant antibody response (RFU ≥ 1000) in SARS-CoV-2-negative (blue) and SARS-CoV-2-positive (red) groups are depicted. Some peptides are present in both groups, shown as overlap.

Figure S2. Relationship between structural properties of epitope sites residues, related to Figure 3. (A) Comparison of solvent accessibility (SASA) between residues in differential epitope sites and rest of the protein. The group "Epitope_AA" includes all residues that are part of differential epitopes and was compared with rest of the amino acids in the protein ("Others"). Student t-test was performed (ns = not significant, * = <0.05). (B) Correlation plot between RFU, SASA and, conservation score (Cscore) in differential epitope sites. Top triangle above the diagonal shows p-value between correlations (*** = > 0.001, **= > 0.01, * = > 0.05). Bottom triangle shows the correlation coefficients. (C) Correlation between conservation score and average solvent accessibility in differential epitope sites.

Figure S3. Identified differential epitope sites in ORF1A of SARS-CoV-2, related to Figure 3. Relative Fluorescence Unit (RFU) values of HDPA analysis were used to calculate ratio values to define differential epitope sites and are color coded. Residues that are not part of epitopes are marked in grey.

Figure S4. Identified differential epitope sites in ORF1B of SARS-CoV-2, related to Figure 3. Relative Fluorescence Unit (RFU) values of HDPA analysis were used to calculate ratio values to define differential epitope sites and are color coded. Residues that are not part of epitopes are marked in grey.

Figure S5. Sensitivity analysis of the number of cross-reactive epitope sites that define a cross-reactive epitope, related to Figure 5. (A) Numbers of cross-reactive epitopes per patient

in relation to the number of cross-reactive epitope sites in HDPA (blue) and recently published PhIP-Seq study (red; [30]). (B) Slope of the correlation between the average antibody response and the number of cross-reactive epitopes in relation to the minimum number of cross-reactive epitope sites in HDPA (blue) and recently published PhIP-Seq study (red; [30]). Using HDPA, the antibody responses were measured as relative fluorescent units (RFU), while it was measured as the Z-score in a recently published PhIP-Seq study [30]. Data have been normalized before performing the linear regressions to respect the assumption of normally distributed residues.

Figure S6. Evolution profile of cross-reactive epitope sites compared to the global epitope pool, related to Figure 6. (A) Distribution of the within-host mutation rates of cross-reactive epitope sites (orange) vs non-cross-reactive epitope sites (grey) across samples is shown. (B) Distribution of the substitution rate of cross-reactive epitope sites (orange) vs non-cross-reactive epitope sites (grey) across samples is shown. (C) Distribution of pN/pS of cross-reactive epitope sites (orange) vs non-cross-reactive epitope sites (grey) across samples is shown. (D) Distribution of dN/dS of cross-reactive epitope sites (orange) vs non-cross-reactive epitope sites (grey) across samples is shown.

Figure S7. Evolutionary profile of SARS-CoV-2 epitopes during the second pandemic wave, related to Figure 6. The extent to which natural selection for immune evasion acts on SARS-CoV-2 during infection, or upon transmission is investigated. The distribution of evolutionary parameters in epitope sites (orange) vs non-epitope sites (grey) during the second pandemic SARS-CoV-2 wave (defined as August 1 to December 31, 2020) is depicted. For each metric, significantly lower values in epitope sites of a certain gene are represented by a blue arrow pointing down while significantly higher values in epitope sites of a certain gene are represented by a red arrow pointing up (FDR-adjusted Wilcoxon test $p<0.05$). (A) Distributions of sample mutation rates (*log10(Count/gene length + 1e-6)*) across targeted proteins during were analyzed. (B) Distributions of sample substitution rate (*log10(Count/gene length + 1e-6)*) across targeted proteins were investigated. (C) Analysis of distributions of sample pN/pS across targeted proteins. (D) Distributions of sample dN/dS across targeted proteins were examined.

Fig. S8. Density of nonsynonymous signature mutations of SARS-CoV-2 variants located at epitope sites, related to Figure 7. Density of nonsynonymous signature mutations in epitopes of selected VOCs and VUIs normalized by gene length. For each VOC/VUI we indicate the density of signature mutations in epitopes identified with HDPA across all analyzed ORFs: envelope (E) protein (orange), membrane (M) glycoprotein (grey), N sites overlapping ORF9c (black), ORF1b (blue), nucleocapsid (N) phosphoprotein (purple), ORF1A (red), and spike (S) protein (green).

Table S1. Serum Samples and Study Population, related to STAR Methods.
Positivity of SARS-CoV-2 infection was assessed both by PCR.

Table S12. Coronavirus taxonomy and sequence accession numbers for analyzed proteins, related to STAR Methods.
